# How continuous is continuous enough? Comparing the reliability of continuous and discrete scales

**DOI:** 10.3758/s13428-026-03080-3

**Published:** 2026-06-17

**Authors:** Wei-Hung Yang, Yao-Ting Sung, Yeh-Tai Chou

**Affiliations:** 1 Department of Educational Psychology and Counseling, National Taiwan Normal University, 162, Sec. 1, Ho-Ping E. Road, Taipei, 10644 Taiwan; 2 Research Center for Psychological and Educational Testing, National Taiwan Normal University, Taipei, Taiwan

**Keywords:** Rating scales, Response granularity, Reliability, Visual analogue scale (VAS), Continuous response model, CoRSA

## Abstract

Designing a reliable rating scale with an appropriate number of options remains a central issue in social science measurement. Prior studies have reported inconsistent findings, with some suggesting that reliability is maximized at moderate response granularity and others indicating that finer granularity yields higher precision. These inconsistencies may partly reflect methodological differences, particularly the use of classical test theory or the discretization of continuous responses, both of which may underestimate the reliability of continuous scales. To address these issues, the present study reconceptualized discrete and continuous scales as points along a unified granularity continuum and examined reliability using the continuous rating scale model (CoRSM). Utilizing the CoRSA analytical framework (Chou et al., 2025), we conducted two complementary studies. Study 1 employed Monte Carlo simulations varying sample sizes (*N* = 200 to 1,000), test lengths (11 to 61 items), and response formats ranging from three-point to continuous. Study 2 provided empirical validation with 3,434 junior high school students completing a career interest assessment across 10 response formats, ranging from five-point scales to continuous visual analogue scales (VAS). Across both studies, reliability generally increased as response granularity increased, although the empirical pattern was not strictly monotonic across all intermediate formats. In Study 2, segmented regression indicated that the breakpoint in diminishing returns occurred at approximately seven to eight response options within the discrete range examined. Meanwhile, the highest reliability estimates were observed for the 101-point and VAS formats. These findings clarify one methodological source of inconsistency in prior research.

## Introduction

### Importance of rating scales

Rating scales are among the most widely used tools in psychology, education, and survey research for quantifying attitudes, perceptions, and other latent traits (Kutscher & Eid, 2024; Preston & Colman, 2000; Uher, 2022). The reliability and precision of measurements obtained from rating scales are crucial, as these instruments inform decision-making in both research and practical assessment contexts. However, empirical studies have shown that the number of response options in a rating scale is associated with variations in reliability, validity, and measurement precision, thereby affecting the overall quality of the measurement (Aybek & Toraman, 2022; Kusmaryono et al., 2022). Too few options may reduce the scale’s ability to discriminate between respondents, while too many may increase cognitive load and response error. Therefore, optimizing the number of response options is crucial for improving scale quality and ensuring valid and reliable measurement outcomes.

### Historical development of rating scales

The earliest form of rating scales can be traced back to the visual analogue scale (VAS). Hayes and Patterson (1921) first applied the VAS to assess psychological states. The VAS consists of a horizontal line segment with opposing semantic anchors (e.g., “strongly agree” vs. “strongly disagree”) at each end. Participants indicate their feelings by marking a point along the line, and the score is calculated as the proportional distance from one endpoint (Cook et al., 2001; Couper et al., 2006). However, before the advent of computers, the VAS was difficult to implement because each mark had to be measured manually with a ruler, which made the process time-consuming and prone to error (Kersten et al., 2014).

In 1932, Rensis Likert introduced the Likert scale as a crucial tool for assessing psychological traits and attitudes. The original Likert scale was a symmetrical five-point scale offering equidistant semantic response options, such as “strongly agree,” “agree,” “neutral,” “disagree,” and “strongly disagree” (Likert, 1932). Because of its simple structure and ease of scoring and analysis, the Likert scale gradually became popular and was later expanded into six-, seven-, nine-, and 11-point variants.

Conceptually, the key difference between the VAS and the Likert scale lies in their response granularity. The Likert scale consists of countable categorical responses that produce discrete scores and therefore represent a coarse-grained measurement tool (Nunnally & Bernstein, 1994; Sung & Wu, 2018). In contrast, the VAS provides a theoretically infinite continuum of possible responses, yielding fine-grained continuous scores (Mellenbergh, 1994; Samejima, 1973; Sung & Wu, 2018).

The digitalization of psychological measurement has eliminated most of the practical difficulties that once limited the use of the VAS. Today, continuous responses can be automatically recorded and precisely scored, making such scales much easier to administer and analyze (Haslbeck et al., 2025; Simms et al., 2019; Sung & Wu, 2018). Because these technical barriers have been resolved, it has become possible to reexamine the advantages of continuous scales under modern testing conditions. As a result, researchers have renewed their interest in whether continuous rating scales provide higher reliability and measurement precision than discrete scales (Haslbeck et al., 2025).

Although the design of rating scales can influence several aspects of measurement quality, including validity, response bias, and respondent burden, the present study focuses specifically on measurement reliability, because reliability provides a fundamental indicator of measurement precision across different rating scale formats. Previous research has shown that rating scale formats may affect measurement outcomes through multiple mechanisms. For example, scales with a large number of response categories may be more susceptible to response styles such as extreme responding or acquiescence (Baumgartner & Steenkamp, 2001; Van Vaerenbergh & Thomas, 2013). In addition, increasing the number of response options may impose greater cognitive demands on respondents and increase respondent burden (Krosnick & Presser, 2010; Preston & Colman, 2000). These factors may introduce additional sources of systematic measurement error and contribute to variability in empirical findings across studies.

In addition to these substantive factors, methodological aspects of how rating-scale data are analyzed may also influence reliability estimates. In many studies, continuous response formats such as visual analogue scales are analyzed using statistical models originally developed for discrete response categories (Kersten et al., 2014; Simms et al., 2019). As a result, continuous responses are often transformed into a limited number of categories before analysis. This discretization process may alter the distribution of observed responses and reduce variability in the data, which can in turn affect the estimation of reliability. Consequently, differences in analytical frameworks may partly contribute to the inconsistent conclusions reported in previous studies regarding the relationship between response granularity and measurement reliability.

Recent developments in continuous-response modeling provide new tools for addressing this methodological issue. In particular, the continuous rating scale analytics (CoRSA; Chou et al., 2025) allows responses recorded on a continuous scale to be modeled directly within an item response theory framework without requiring the continuous response continuum to be manually partitioned into discrete response categories prior to analysis (Kersten et al., 2014; Simms et al., 2019). By estimating person ability and measurement error from continuous responses, this approach enables researchers to examine how response granularity influences reliability within a unified measurement framework that treats discrete and continuous scales as points along the same continuum. These methodological advances make it possible to revisit the long-standing debate regarding how response granularity influences measurement reliability using a unified analytical approach.

### The debate on how rating scale granularity influences measurement reliability

Despite the long history of rating scales, researchers still have not reached a consensus on the optimal number of response options that maximize reliability and validity. The debate centers on whether coarse-grained discrete scales (such as five- or seven-point Likert scales) are sufficient, or whether fine-grained and continuous formats yield better psychometric performance.

On one hand, numerous studies have found that a small set of response options can provide sufficient reliability and validity. Early investigations by Symonds (1924) and Bendig (1954) demonstrated that reliability increased as the number of response options increased from two to seven but leveled off beyond that point, suggesting that additional options offered little benefit. Subsequent studies conducted Monte Carlo simulations to compare the reliability of scales with varying numbers of response options and produced similar findings. Lozano et al. (2008) demonstrated that reliability and validity improved substantially as the number of response options increased from two to seven, after which additional options yielded minimal gains. Likewise, Lee and Paek (2014) applied a graded response model under the item response theory framework and identified four to six response options as the most stable and efficient range.

More recent empirical research has provided converging evidence. Preston and Colman (2000) reported that scales with seven to ten options achieved the best balance between reliability and respondent usability, whereas having fewer than five or more than ten options decreased reliability. Similarly, Simms et al. (2019) found that reliability increased up to seven points but did not improve further when more options or a continuous format were used.

Taken together, these findings suggest a general consensus that coarse-grained scales with approximately five to seven response options provide an optimal trade-off between measurement precision and respondent burden. Beyond this range, additional response options yield diminishing returns and may even impair reliability due to increased cognitive load.

On the other hand, some researchers have argued that finer response granularity theoretically expands score variability and allows respondents to express more subtle distinctions in their judgments (Krieg, 1999; Funke & Reips, 2012; Sung & Wu, 2018). Thus, increasing the number of response options can enhance reliability rather than diminish it. For example, Champney and Marshall (1939) found that reliability increased notably as the number of response options rose to 12, with smaller gains observed up to 30 and a slight decline thereafter. Similarly, Garner (1960) tested seven rating scales ranging from four to 20 response options and found that score variability and discriminability increased as the number of options increased. He concluded that there was no single optimal number, but that using more options generally improved measurement precision without loss of information. Krieg (1999) conducted simulation studies within a classical test theory framework and demonstrated that measurement error decreases as the number of response options increases and approaches a minimum as the scale becomes continuous. He also suggested that continuous scales, such as the VAS, may reduce cognitive load because respondents can indicate their exact position on a continuum instead of being constrained to a limited number of options. Furthermore, Funke and Reips (2012) observed that VAS responses correlated more strongly with external behavioral criteria than Likert responses, indicating higher criterion-related validity. Similarly, Sung and Wu (2018) compared a five-point Likert scale with a continuous VAS in a career interest assessment and found higher reliability for the VAS, concluding that finer granularity improves reliability. Taken together, these simulation and empirical studies suggest that fine-grained or continuous scales can provide higher reliability and validity than coarse-grained discrete ones.

Although many studies have examined the relationship between scale reliability and response granularity, the findings remain inconsistent. It is still unclear whether increasing response granularity always leads to better reliability or whether the benefits eventually plateau. This uncertainty highlights the need for a more systematic investigation into how scale granularity influences reliability.

### The unsolved issues of previous studies on reliability of continuous and discrete scales

**Fig. 1:**
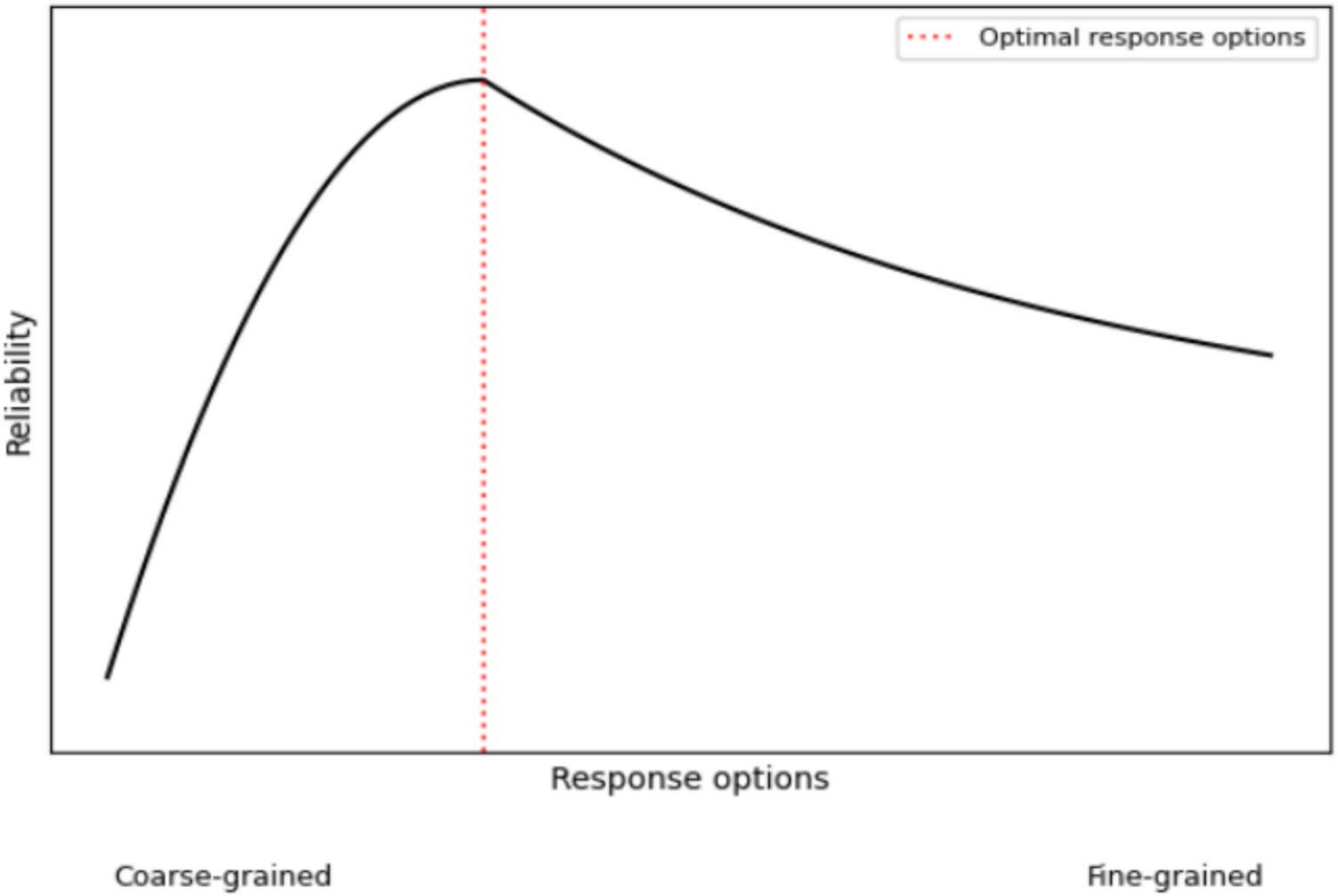
Inverted-U pattern of reliability across response option numbers

When synthesizing previous evidence, two general patterns can be observed in describing the relationship between scale reliability and response granularity. The first is the inverted-U pattern, as shown in Fig. 1. In this pattern, reliability increases as the number of response options increases but declines after reaching an optimal range, typically around six to seven points (Bendig, 1954; Lee & Paek, 2014; Lozano et al., 2008; Preston & Colman, 2000; Simms et al., 2019; Symonds, 1924). As a result, an optimal number of response options can be identified across different levels of granularity. Furthermore, increasing response granularity does not always lead to higher reliability; in other words, continuous scales do not necessarily produce the greatest reliability.

**Fig. 2:**
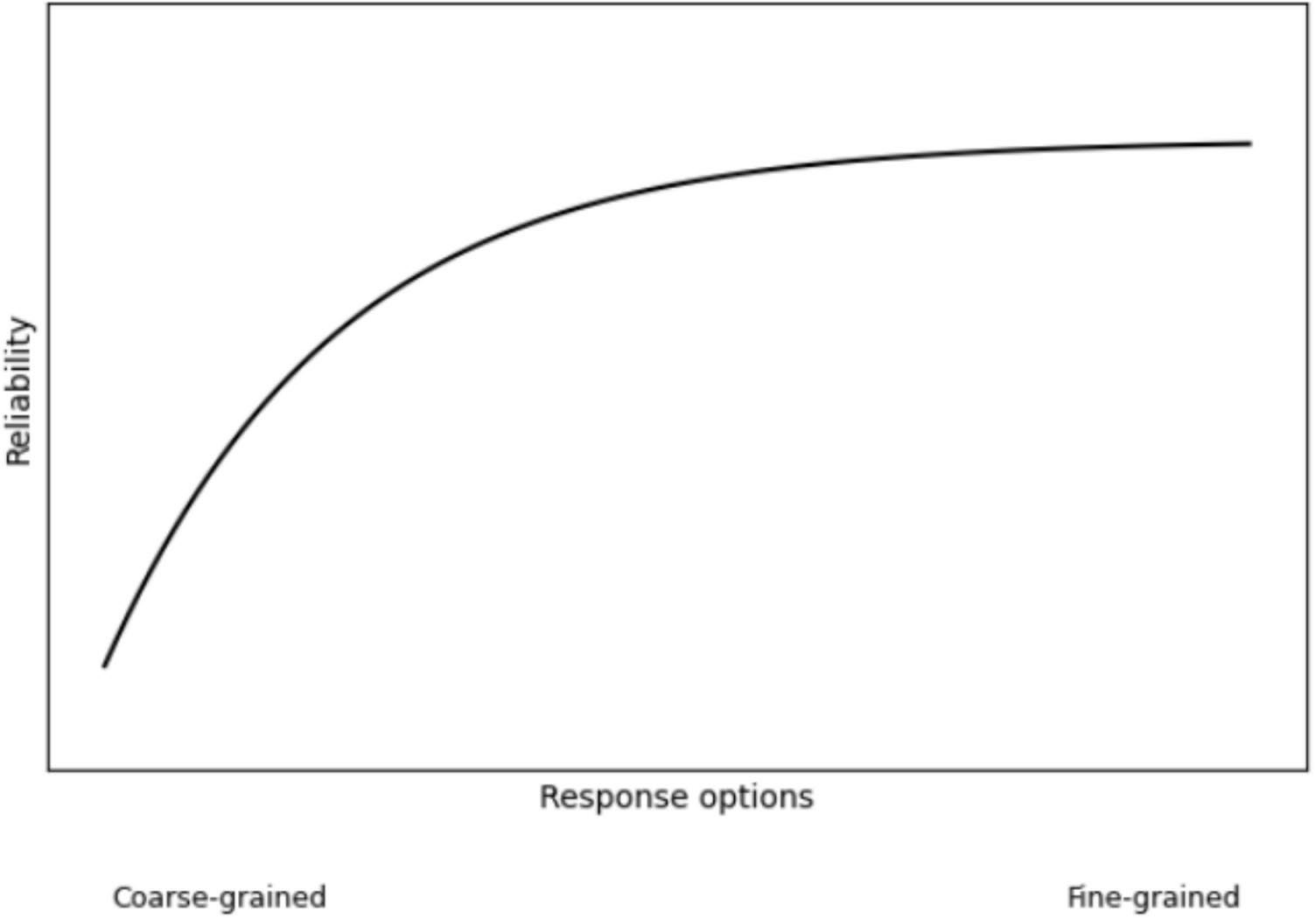
Monotonically increasing pattern of reliability across response option numbers

The second pattern is a monotonically increasing trend, as shown in Fig. 2. In this pattern, other studies have shown that reliability continues to gradually increase as more options are added, suggesting that finer granularity may continuously improve measurement precision (Champney & Marshall, 1939; Funke & Reips, 2012; Garner, 1960; Krieg, 1999; Sung & Wu, 2018). As a result, the reliability of continuous scales would always be higher than that of discrete scales. Notably, the incremental gain in reliability between scales with adjacent levels of granularity decreases as granularity increases, implying that the effect of adding one more response option on improving reliability diminishes.

These inconsistencies may arise from multiple sources, including both substantive and methodological factors. One major methodological issue concerns the analytical framework used to estimate reliability. Many earlier studies relied on classical test theory (CTT; Preston & Colman, 2000; Simms et al., 2019). However, Kim and Feldt (2010) showed that CTT tends to underestimate reliability when compared with item response theory (IRT). As a result, studies using CTT to evaluate rating scales with different levels of response granularity may have underestimated their true reliability.

To avoid the aforementioned problem of underestimating scale reliability, some studies adopt IRT to analyze rating scale data. However, researchers have faced significant challenges when analyzing continuous-response data. Because most IRT models were developed for discrete data, continuous data are often discretized before analysis. For example, Kersten et al. (2014) measured participants’ pain intensity using a visual analogue scale (VAS) and divided the continuous line into 51 discrete categories before applying the partial credit model (Masters, 1982). Similarly, Simms et al. (2019) used a VAS and divided the 1,000-point continuum into equal segments to compute reliability estimates based on discretized scores. Such discretization can substantially distort measurement properties. Rucker et al. (2015) demonstrated that dichotomizing continuous variables using the median-split procedure drastically reduces individual-level variance, since respondents with different true scores may be grouped into the same category. In CTT, reliability is defined as the ratio of true-score variance to observed-score variance; when observed variance decreases, reliability inevitably declines. MacCallum et al. (2002) further confirmed through simulation that dichotomizing continuous data can reduce reliability from 0.80 to 0.50. A decrease in reliability implies greater measurement error, which weakens correlations and reduces statistical power (Humphreys & Drasgow, 1989).

A related challenge is the limited availability of analytical tools capable of handling continuous data directly. Although several continuous-response models have been proposed, such as the continuous response model (CRM; Samejima, 1973), the beta response model (BRM; Noel & Dauvier, 2007), and the continuous rating scale model (CoRSM; Müller, 1987), software for applying these models has traditionally been scarce. For example, Müller’s CoRSM was introduced in 1987 to analyze continuous VAS data, yet practical implementations only became available decades later (Chou et al., 2025; Hohensinn, 2018). The lack of accessible tools forced researchers to rely on discretization and ordinal models, which may have contributed to the inconsistent conclusions observed across prior studies.

Beyond these methodological limitations, prior research also lacks a coherent theoretical framework for understanding how scale granularity influences measurement reliability. Most studies have treated discrete and continuous scales as categorically distinct, assuming they represent fundamentally different forms of measurement. In contrast, we argue that response granularity should be conceptualized as a continuous property. From this perspective, coarse-grained and fine-grained scales represent different positions along the same underlying continuum (Chou et al., 2025; Samejima, 1973). Discrete scales can be viewed as special cases of continuous scales, created by imposing artificial thresholds on an otherwise unbroken response continuum. This conceptual clarification enables researchers to investigate how reliability changes incrementally as scale granularity increases, rather than comparing two arbitrarily defined types of scales. It also provides a unifying framework for interpreting the mixed results observed in prior research.

Addressing these issues requires analytical tools capable of modeling continuous responses directly and a unified framework for examining how reliability changes across different levels of scale granularity. To this end, the present study seeks to provide an empirical and simulation-based investigation of the relationship between response granularity and measurement reliability.

### Purpose of this research

To further explore the methodological and conceptual issues identified above, the present research aims to clarify how rating scale granularity affects measurement reliability within the IRT framework. Unlike previous research that relied on classical test theory or discretized continuous data for IRT analysis, this study applies modern continuous-response modeling techniques that can directly estimate reliability from continuous responses. By using these tools, we aim to overcome the analytical limitations that have hindered prior comparisons between discrete and continuous rating formats.

Specifically, this research addresses two unresolved questions. The first question concerns the functional relationship between scale granularity and measurement reliability. We examine whether reliability first increases and then declines after reaching an optimal range, or whether it continues to increase steadily as the number of response options grows, suggesting that finer and continuous scales yield progressively higher reliability. The second question focuses on practical equivalence. If continuous scales represent the upper limit of reliability, how many response options are needed for a discrete scale to approximate the reliability of a continuous one? Identifying this point of equivalence can provide an empirical guideline for determining the optimal number of response options.

To address these questions, we conduct two complementary studies. Study 1 employs systematic simulation analyses to compare the reliability of discrete scales with varying numbers of response options with that of continuous scales. Utilizing the continuous rating scale analytics (CoRSA; Chou et al., 2025) framework, an analytical approach for estimating person ability and reliability from continuous-response data, we examine whether a monotonic relationship exists between scale granularity and reliability. If such a relationship is observed, we further examine how many response options are needed for a discrete scale to approximate the reliability of a continuous scale. Study 2 analyzes empirical data to test whether the simulation findings generalize to real measurement contexts. This study aims to validate the simulation results using actual response data collected from applied settings.

By integrating theoretical reasoning with both simulation and empirical evidence, this study aims to clarify how response granularity influences measurement reliability within a continuous-response modeling framework and to provide empirical guidance for selecting appropriate numbers of response options for reliable and efficient measurement.

## Study 1: Using simulated data to examine the relationship between reliability and response granularity

Study 1 examined how measurement reliability changes as the number of response options increases under different measurement conditions. We evaluated whether reliability increases monotonically with response granularity and whether such improvements diminish as the scale approaches continuity.

### Method

A series of Monte Carlo simulations were conducted using data generated from the CoRSM (Müller, 1987). The CoRSM is an extension of the rating scale model (RSM; Andrich, 1978) designed for situations in which responses are recorded on a continuous scale rather than as discrete categories. In the CoRSM framework, the rating scale is represented as a bounded continuum on which respondents can indicate any point within the interval. Consequently, the observed response is modeled as a continuous variable whose probability density is determined by the difference between person ability and item difficulty. Responses are assumed to follow a truncated normal distribution along the response continuum. Unlike traditional IRT models that specify probabilities for selecting discrete response categories, the CoRSM models the density of responses across the entire continuum, allowing both continuous responses (e.g., visual analogue scales) and discretized rating categories (e.g., Likert-type scales) to be analyzed within the same framework.

Let *x*_*ni*_ refer to a continuous response of person *n* responding to item *i*. The probability density of *x*_*ni*_ is expressed as follows:

1 $$f\left(X={x}_{ni}\left|{\beta}_{n},{\delta}_{i},\theta \right.\right)=\frac{\mathrm{exp}\left[{x}_{ni}\left({\beta}_{n}-{\delta}_{i}\right)+{x}_{ni}\left(2c-{x}_{ni}\right)\theta \right]}{{\int}_{c-L/2}^{c+L/2}exp\left[t\left({\beta}_{n}-{\delta}_{i}\right)+t\left(2c-t\right)\theta \right]dt}$$

where ${\beta}_{n}$ denotes person ability, ${\delta}_{i}$ denotes item difficulty, *θ* is a dispersion parameter, *L* is the rating scale length, and *c* is the scale midpoint. Simulation procedures were implemented using custom scripts in R statistical software (version 4.1.2; R Core Team, 2018). The R code used for the simulations is publicly available in the Open Science Framework repository (https://osf.io/vq6xg).

#### Design

Three independent variables were manipulated: sample size (200, 500, and 1,000 subjects, representing small, medium-sized, and large samples, respectively), test length (11, 21, 41, and 61 items, representing extremely short, short, medium-length, and long tests, respectively), and the number of response options (3–20 points plus a continuous score condition). Although response scales exceeding 11 points are rarely used in practice, they were included to examine the theoretical asymptotic relationship between granularity and reliability. In total, 228 conditions (3 × 4 × 19) were evaluated with 100 replications each.

On the basis of classical and item response theory, we expected that reliability would increase with test length and greater response granularity, while sample size would have minimal influence on reliability estimates.

#### Data generation

Simulated data were generated following a five-step procedure that implements the CoRSM’s probability density function and numerically samples continuous responses on a bounded rating continuum. The process consisted of the following steps: (a) The ability parameters were randomly drawn from the standard normal distribution, *N*(0, 1). (b) The difficulty parameters were evenly spaced across [–2, 2], and the dispersion parameter was set to 0.5. In the CoRSM, the dispersion parameter controls the shape of the response distribution along the rating continuum (Andrich, 1982). Smaller dispersion values correspond to wider response distributions and greater variability in item responses (Verhelst, 2019). In the present simulation, the dispersion parameter was held constant to represent a moderate level of response dispersion while isolating the effect of response granularity on reliability. (c) The scale length was set to *L* = 6 with midpoint *c* = 0, producing a response range of [–3, 3]. (d) The response range was partitioned into 100 equally spaced subintervals. For each subinterval [*x*_*q*_, *x*_*q*+1_], midpoint values *c*_*q*_ = (*x*_*q*_ + *x*_*q*+1_)/2 were computed and served as candidate response values. Approximate probability mass was computed using Eq. (1), forming a discrete approximation to the continuous probability density function. (e) For each person-item pair, a uniform random value *u* was drawn from *U*(0, 1), representing a random draw used to sample a response from the cumulative probability distribution implied by the CoRSM, thereby generating an observed response consistent with the model. The response *x*_*ni*_ was assigned to the midpoint *c*_*q*_ corresponding to the cumulative probability that equaled or just exceeded *u*. Discrete responses were created by partitioning the continuous range into *m* equal-width categories (where *m* denotes the number of response options). Each continuous *x*_*ni*_ was mapped to the nearest category midpoint to ensure that continuous and discrete data shared the same underlying latent response distribution.

All simulated datasets were calibrated using self-written R functions and codes that implemented the estimation algorithms of the CoRSA (Chou et al., 2025). CoRSA is the analytical framework used to estimate person ability and measurement error from continuous-response data under the CoRSM, thereby allowing both continuous and discretized response formats to be analyzed within a common measurement framework.

#### Data analysis

In each scenario, each simulated dataset was analyzed using CoRSA (Chou et al., 2025), and the following statistics were calculated: (a) the variance of all examinee ability estimates; (b) the mean squared error of all examinee ability estimates (as in Eq. 2); (c) the reliability of ability estimates (e.g., Eq. 3).

2 $${\sigma}_{error}^{2}=\frac{1}{N}\sum_{n=1}^{N}{SEM}_{n}^{2}$$

3 $$\rho =\frac{{\sigma}_{obs}^{2}-{\sigma}_{error}^{2}}{{\sigma}_{obs}^{2}}$$

where ${\sigma}_{obs}^{2}$ is the variance of ability estimates while ${\sigma}_{error}^{2}$ is the mean variance of the ability estimate error.

### Results

**Fig. 3:**
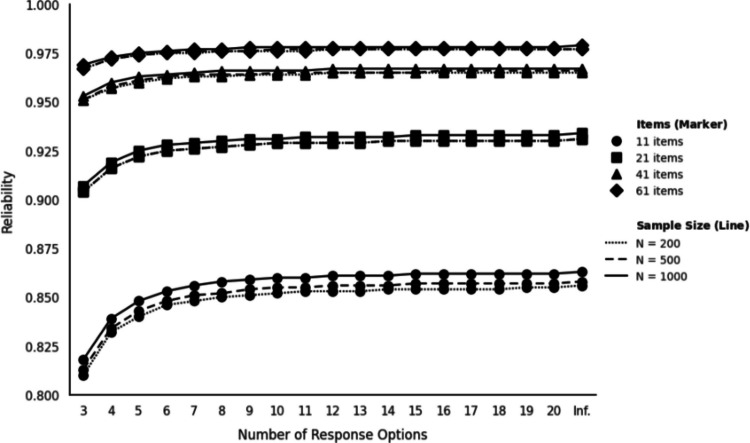
Reliability estimates across numbers of response options

Table 1 summarizes the reliability estimates obtained under different combinations of sample size, test length, and number of response options, with Fig. 3 providing a visual illustration of the overall pattern. Across all conditions, reliability increased as the number of response options increased, although the rate of improvement became progressively smaller as response granularity increased.

**Table 1:** Reliability estimates across test lengths, sample sizes, and numbers of response options

Test length	Sample size	Number of response options																		
		3	4	5	6	7	8	9	10	11	12	13	14	15	16	17	18	19	20	inf
11	200	.810	.832	.840	.846	.848	.850	.851	.852	.853	.853	.853	.854	.854	.854	.854	.854	.855	.855	.856
	500	.813	.834	.843	.848	.851	.852	.854	.855	.855	.856	.856	.856	.857	.857	.857	.857	.857	.857	.858
	1,000	.818	.839	.848	.853	.856	.858	.859	.860	.860	.861	.861	.861	.862	.862	.862	.862	.862	.862	.863
21	200	.904	.916	.922	.925	.926	.927	.928	.929	.929	.929	.929	.930	.930	.930	.930	.930	.930	.930	.931
	500	.904	.916	.922	.925	.926	.927	.928	.929	.929	.929	.929	.930	.930	.930	.930	.930	.930	.930	.931
	1,000	.907	.919	.925	.928	.929	.930	.931	.931	.932	.932	.932	.932	.933	.933	.933	.933	.933	.933	.934
41	200	.951	.957	.960	.962	.963	.963	.964	.964	.964	.965	.965	.965	.965	.965	.965	.965	.965	.965	.965
	500	.951	.958	.961	.963	.964	.964	.964	.965	.965	.965	.965	.965	.965	.966	.966	.966	.966	.966	.966
	1,000	.953	.960	.963	.964	.965	.966	.966	.966	.966	.967	.967	.967	.967	.967	.967	.967	.967	.967	.967
61	200	.967	.972	.974	.975	.975	.976	.976	.976	.976	.977	.977	.977	.977	.977	.977	.977	.977	.977	.977
	500	.967	.972	.974	.975	.976	.976	.976	.977	.977	.977	.977	.977	.977	.977	.977	.977	.977	.977	.977
	1,000	.969	.973	.975	.976	.977	.977	.978	.978	.978	.978	.978	.978	.978	.978	.978	.978	.978	.978	.979

This pattern was especially evident for shorter tests. For example, for the 11-item test with 200 participants, reliability increased from.810 with three response options to .848 with seven response options and .851 with nine response options, whereas the continuous condition yielded .856. Similarly, for the 21-item test with 200 participants, reliability increased from .904 with three response options to .926 with seven response options and .928 with nine response options, reaching .931 in the continuous condition. These results indicate that the largest gains occurred in the lower-to-mid range of response granularity, whereas additional gains at higher levels were comparatively modest.

Longer tests showed the same general pattern but with higher overall reliability and an earlier reduction in marginal gains. For the 41-item test with 200 participants, reliability increased from .951 with three response options to .963 with seven response options and .964 with nine response options, with the continuous condition yielding .965. For the 61-item test with 200 participants, reliability increased from .967 with three response options to .975 with seven response options and .976 with nine response options, reaching .977 in the continuous condition. Thus, although reliability continued to increase with finer-grained response formats, the magnitude of these increases became smaller more quickly as test length increased.

The same general pattern was observed across all three sample size conditions, indicating that sample size had little influence on the shape of the reliability–granularity relation under the present simulation settings. Overall, the simulated data suggest that reliability tends to improve as response scales become finer-grained, but that these improvements exhibit diminishing returns. In addition, the results indicate a compensatory relation between test length and response granularity: longer tests reached a near-asymptotic level of reliability with fewer response options, whereas shorter tests showed greater benefit from increases in response granularity. Table 1 also shows that reliability increased systematically with test length when the number of response options was held constant, consistent with general expectations from classical test theory and item response theory.

## Study 2: Using empirical data to examine the relation between reliability and response granularity

Study 2 examined how reliability varied across response formats with different levels of granularity in an empirical assessment context. Building on the simulation results from Study 1, this study evaluated whether reliability generally increased as response granularity increased, whether the rate of improvement diminished within the discrete response range, and how closely highly fine-grained discrete formats approximated the reliability of a continuous visual analogue scale (VAS). In addition, a formal breakpoint analysis was conducted to identify where marginal reliability gains began to diminish in the discrete response range.

### Method

#### Assessment tools and data collection

The sample for this study consisted of 3,434 Taiwanese junior high school students in the eighth and nineth grades, including 1,789 male and 1,645 female students, with an average age of 13.7 years. These students participated in a situation-based career interest assessment (SCIA; Sung et al., 2016, 2017) for the purpose of streaming into senior high schools or vocational high schools. The parents of all the students had already given their consent for their children to take part in this research prior to the data collection.

**Fig. 4:**
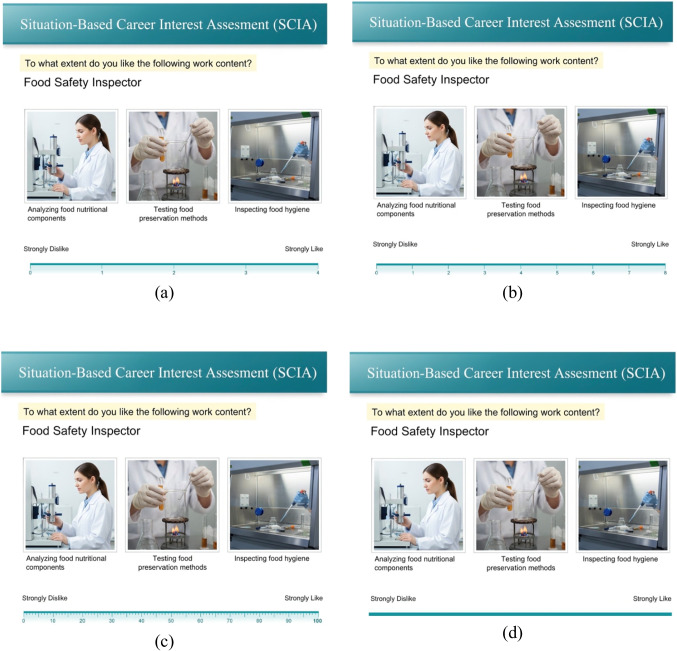
Examples of four different response option formats used in the SCIA items: **a** five-point scale, **b** nine-point scale, **c** 101-point scale, and **d** visual analogue scale (VAS)

The SCIA (Sung et al., 2016, 2017) is a situational computerized interest test tool developed based on Holland’s (1997) theory of career choice. Utilizing information and multimedia technology, SCIA is designed to assist students in grades 7 to 9 in making career choices. Career interests are categorized into six types, according to Holland’s theory: realistic (R), investigative (I), artistic (A), social (S), enterprising (E), and conventional (C). In this study, we employed a simplified version of SCIA (see Fig. 4), designing 10 items for each type, resulting in a total of 60 items across the test. Each item assesses the students’ preference levels using measurement tools with varying numbers of response options. The right side of the scale indicates an increase in preference, while the left side signifies an increase in aversion. After reading the items, students can move the icons to any position on the scale they deem appropriate, and these positions are used for subsequent scoring. Before taking the formal test, students complete a practice test.

#### Linking design

To examine how reliability varied across response formats with different levels of granularity in an empirical assessment context, an overlapping linking design was used (Oosterveld et al., 2019; van der Linden & Ren, 2015; Zijlmans et al., 2019). This design allowed response formats administered in different groups to be connected through shared formats and placed on a comparable measurement continuum.

Participants were assigned to experimental conditions at the classroom level because the assessment was administered in school settings. Thus, all students within the same classroom completed the same response-format condition, and each student participated in only one experimental group.

Eight experimental groups were formed. The response-format combinations followed a granularity ladder in which adjacent groups shared one overlapping format. This structure enabled local linking across increasingly fine-grained response formats, from coarse discrete scales to very fine-grained discrete scales and, finally, to a continuous format. In most groups, students completed two response formats on the same set of items. Only the first group completed three formats because a longer administration time was available during the initial phase of data collection. The eight response-format combinations were as follows: (1) 5-, 7-, and 9-point scales; (2) 9- and 10-point scales; (3) 10- and 11-point scales; (4) 11- and 12-point scales; (5) 12- and 13-point scales; (6) 13- and 20-point scales; (7) 20- and 101-point scales; and (8) 101-point and VAS formats. Through these overlapping formats, reliability estimates obtained from different groups could be compared along a linked granularity continuum. All response formats assigned to a given group were completed within the same testing session. Table 2 presents the response-format combinations and sample sizes for the eight experimental groups.

**Table 2:** Response-format combinations and sample sizes for the eight experimental groups

Group	*N*	5	7	9	10	11	12	13	20	101	VAS
1	371	V	V	V							
2	562			V	V						
3	479				V	V					
4	423					V	V				
5	567						V	V			
6	652							V	V		
7	380								V	V	
8	432									V	V

V indicates that the corresponding response format was administered in the given experimental group

#### Analysis

Analyses were conducted separately for each RIASEC dimension (R, I, A, S, E, and C), with each dimension modeled independently on the basis of its corresponding 10 items. As an initial check on the overlapping linking design, Levene’s tests were conducted for response formats shared across adjacent experimental groups. These shared formats served as linking anchors in the overlapping design. Across these comparisons, Levene’s tests were nonsignificant (all *p*s >.05), suggesting that the assumption of homogeneity of variance was not violated for groups sharing the same response format.

The empirical response data were then analyzed using the same continuous-response analytical framework as in Study 1. Specifically, CoRSA (Chou et al., 2025) was used to estimate person ability and its associated measurement error for each participant. On the basis of these outputs, reliability was computed for each response format and each RIASEC dimension using the variance of the ability estimates and the mean squared standard errors of measurement.

To identify where marginal gains in reliability began to diminish, an additional breakpoint analysis was conducted using segmented regression in R (Muggeo, 2003, 2008), which provides a direct estimate of the point at which the slope of the reliability–granularity relationship changes. The primary breakpoint analysis focused on the discrete response formats from 5 to 20 response options because these formats represent the practically relevant range for discrete scale design and allow the onset of diminishing returns to be evaluated without conflating discrete and continuous response regimes. A robustness analysis was then conducted by extending the discrete range to include the 101-point format. The VAS condition was not included in the primary breakpoint model because it yielded continuous responses on a 0–1 continuum rather than a fixed number of discrete response categories. Instead, the VAS results were retained as a continuous-format benchmark for descriptive comparison.

For the segmented regression analyses, reliability was modeled as a function of response granularity, and a single breakpoint was estimated to identify where the slope of the reliability–granularity relation changed. Breakpoint analyses were conducted both for each RIASEC dimension separately and for the mean reliability curve across dimensions. In addition, Davies tests were used to evaluate whether the change in slope was statistically supported. The segmented regression analyses were implemented in R, and the corresponding analysis scripts are publicly available in the Open Science Framework repository (https://osf.io/vq6xg).

### Results

**Fig. 5:**
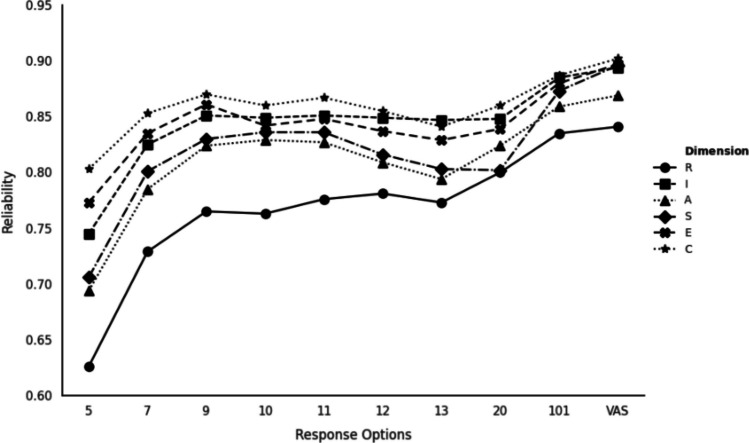
Reliability values under different response options across six dimensions

Table 3 and Fig. 5 summarize the empirical reliability estimates across response formats for the six RIASEC dimensions. Overall, reliability generally increased as response scale granularity increased, although the pattern was not strictly monotonic across all response formats.

**Table 3:** Reliability values under different response options across six dimensions

Response options	*N*	R	I	A	S	E	C
5	371	.626	.745	.694	.706	.773	.803
7	371	.729	.825	.785	.801	.835	.853
9	371	.765	.851	.824	.830	.861	.870
10	562	.763	.849	.829	.836	.842	.860
11	1041	.776	.851	.827	.836	.848	.867
12	902	.781	.849	.809	.816	.837	.855
13	878	.773	.847	.794	.803	.829	.841
20	966	.800	.848	.824	.802	.839	.860
101	891	.835	.885	.859	.873	.880	.887
VAS	380	.841	.894	.869	.896	.897	.902

R = realistic; I = investigative; A = artistic; S = social; E = enterprising; C = conventional

To formally examine where marginal gains in reliability began to diminish, a segmented regression analysis was conducted using the discrete response formats from 5 to 20 points. For the mean reliability curve across the six dimensions, the estimated breakpoint was 7.620 response options (*SE* = 0.302). A robustness analysis extending the discrete range to include the 101-point format yielded a highly similar breakpoint estimate of 7.515 (*SE* = 0.274). Dimension-specific breakpoint estimates ranged from 7.372 to 7.755 response options (Table 4), indicating a highly consistent pattern across the six dimensions. Davies tests supported the presence of a statistically significant change in slope for the mean curve and for each dimension individually.

**Table 4:** Breakpoint estimates from segmented regression analyses

Dimension	Breakpoint	*SE*	Davies test *p*-value
R	7.617	0.166	<.001
I	7.633	0.055	<.001
A	7.755	0.524	0.008
S	7.723	0.343	0.002
E	7.453	0.504	0.014
C	7.372	0.602	0.032
Mean (5–20)	7.620	0.302	0.001
Mean (5–101)	7.515	0.274	Not applicable

Breakpoints represent estimated locations at which the slope of the reliability–granularity relation changes. Davies tests evaluate whether a significant change in slope is present. Breakpoints for the primary analysis were estimated using discrete response formats from 5 to 20 options. The extended analysis including the 101-point format was conducted as a robustness check

Consistent with these breakpoint estimates, the descriptive results showed comparatively large increases in reliability at lower levels of granularity, particularly between five and seven response options, with additional but smaller gains from seven to nine response options. Across the intermediate discrete formats from 9 to 20 points, reliability values fluctuated within a relatively narrow range, indicating a substantial reduction in the rate of improvement relative to the earlier part of the curve. To illustrate this pattern (see Fig. 5), the R and C dimensions are presented as representative examples because they exhibited the lowest and highest overall reliability, respectively. For the R dimension, reliability increased from.626 at five points to .729 at seven points and .765 at nine points. Across the subsequent discrete formats from 10 to 20 points, the values varied within a relatively narrow range, from .763 to .800, before increasing to .835 at 101 points and .841 in the VAS condition. For the C dimension, reliability increased from .803 at five points to .853 at seven points and .870 at nine points. From 10 to 20 points, the values fluctuated modestly between .841 and .867, followed by further increases to .887 at 101 points and .902 in the VAS condition. The remaining four dimensions, I, A, S, and E, showed the same general pattern, with larger gains at lower levels of granularity and a clear reduction in marginal improvements beginning at approximately seven to eight response options. At higher levels of granularity, reliability continued to increase, but at a slower rate, with the highest estimates observed for the 101-point and VAS formats.

Taken together, these findings indicate that reliability improved as response scales became more fine-grained, but that the rate of improvement diminished substantially after approximately seven to eight response options within the discrete range examined here. Importantly, no single discrete response format was identified as universally optimal, and very fine-grained formats continued to yield the highest reliability estimates.

## General discussion

Rating scale design remains a central issue in psychological measurement. Although many studies have examined how the number of response options influences measurement reliability, their findings have been inconsistent. Some studies suggest that increasing the number of response options enhances reliability up to an optimal point, typically around six or seven options, after which reliability begins to decline (Bendig, 1954; Lee & Paek, 2014; Lozano et al., 2008; Preston & Colman, 2000; Simms et al., 2019; Symonds, 1924). Others, however, report that reliability continues to increase as more response options are added (Champney & Marshall, 1939; Funke & Reips, 2012; Garner, 1960; Krieg, 1999; Sung & Wu, 2018).

These inconsistencies may partly reflect methodological and conceptual factors. First, many earlier studies relied on statistical methods that tend to underestimate the reliability of continuous data, particularly when continuous scores are artificially grouped into categories for analysis. Second, most research has treated discrete and continuous scales as categorically distinct rather than recognizing them as points along a single continuum of response granularity.

To address these issues, this study used CoRSA (Chou et al., 2025), a continuous-response modeling tool built on the IRT framework, to estimate person ability and standard errors directly from continuous responses rather than converting them into discrete categories. This approach enabled a statistically rigorous comparison of reliability across scales with different response options. Using both simulated and empirical data, the study provides a more precise understanding of how response granularity relates to reliability.

The study addressed two core research questions. First, does measurement reliability increase with greater response granularity, or do the gains begin to diminish at higher levels of granularity? Second, if continuous scales provide the highest reliability, how should the practical meaning of discrete response granularity be interpreted relative to continuous formats?

### Does measurement reliability increase with greater response granularity, or does it reach an optimal point?

Across both the simulation and empirical studies, scales with a greater number of response options generally produced higher reliability. However, the gains became progressively smaller as the number of response options increased, and the highest estimates were observed under highly fine-grained or continuous conditions. This general pattern was observed across both studies, although the empirical results were not strictly monotonic across all intermediate formats.

In the simulation study, reliability increased steadily as the number of response options grew, but the rate of improvement declined markedly as granularity increased, approaching an upper bound under the continuous condition. In the empirical study, reliability also increased overall with response granularity, but the pattern was not strictly monotonic across all intermediate formats. The segmented regression analysis indicated that the breakpoint in diminishing returns occurred at approximately seven to eight response options, with the mean breakpoint estimated at 7.620 in the primary analysis and 7.515 in the robustness analysis including the 101-point format.

In addition to response granularity, the simulation results also indicated that reliability was influenced by test length. Specifically, Study 1 showed that longer tests approached a near-asymptotic level of reliability with fewer response categories, suggesting a compensatory relationship between scale length and response granularity. Accordingly, the empirical results observed in Study 2 should be interpreted in light of this relationship.

In comparison with previous research, our results suggest that reliability tends to increase as the number of response options increases under continuous-response modeling conditions, while the marginal gains become smaller beyond the early part of the curve. This finding aligns with studies favoring fine-grained scales (Champney & Marshall, 1939; Funke & Reips, 2012; Garner, 1960; Krieg, 1999; Sung & Wu, 2018) and also helps explain why earlier studies often identified a plateau after a moderate number of response options (Bendig, 1954; Lee & Paek, 2014; Lozano et al., 2008; Preston & Colman, 2000; Simms et al., 2019; Symonds, 1924). This discrepancy is likely attributable to methodological advancements. Whereas previous studies often underestimated reliability by applying classical test theory or discretizing continuous data, which is known to reduce variance and underestimate reliability (MacCallum et al., 2002; Rucker et al., 2015), the present study utilized CoRSA to model continuous responses directly (Chou et al., 2025).

The advantages of finer-grained and continuous response formats can be understood from both a psychometric and cognitive perspective. First, from a psychometric standpoint, discretizing intrinsically continuous traits into limited options fails to capture subtle differences, blurring the true level of the trait. This information coarsening reduces the variance of observed scores, thereby weakening estimates of reliability and covariance (Krieg, 1999). In contrast, continuous scales possess higher response granularity, enabling the capture of minute inter-individual differences. This results in lower measurement error and theoretically approximates true values more closely (Sung & Wu, 2018). Second, from a cognitive perspective, discrete options compel participants to round their actual perceptions to the nearest category, thereby introducing additional decision noise (Funke & Reips, 2012). Continuous scales, however, allow participants to express their psychological intensity more directly and precisely. Through a finer range of response categories, internal states can be mapped more completely onto external metrics. This approach not only preserves complete response information but also avoids the reduction in statistical power caused by scale coarsening (MacCallum et al., 2002).

This rigorous approach suggests that the apparent “decline” in reliability for high-granularity scales reported in prior literature may partly reflect analytical limitations rather than a true psychometric property. Taken together, our findings indicate that finer-grained and continuous response formats can yield higher reliability estimates, although the incremental gains diminish substantially after approximately seven to eight response options within the discrete range examined here.

### If continuous scales provide the highest reliability, how many response options are required for a discrete scale to achieve a comparable level of precision?

Having established that continuous scales provided the highest reliability, the next question concerns how discrete response granularity should be interpreted in practice relative to continuous formats.

The results from the two studies suggest that the largest reliability gains occur at lower-to-mid levels of granularity, whereas additional gains become progressively smaller at higher levels. In the simulation results, moving from lower to moderate numbers of response options produced clear gains in reliability, whereas additional gains became progressively smaller as granularity increased further and reliability approached an upper bound under the continuous condition. In the empirical study, the segmented regression analysis indicated that the breakpoint in diminishing returns occurred at approximately seven to eight response options. At the same time, the observed reliability values showed that very fine-grained formats continued to yield additional gains, with the 101-point and VAS conditions producing the highest estimates, while the difference between them was comparatively small.

Taken together, these findings indicate that continuous response formats tended to yield the highest reliability in the present analysis. However, in practical contexts where discrete scales are preferred or required, our results suggest that discrete response formats beginning around seven to eight response options tend to capture a substantial proportion of the reliability obtainable under more fine-grained and continuous conditions. Although the highest estimates were still observed for the 101-point and VAS formats, scales in this range may provide a practically efficient compromise while retaining the familiar advantages of discrete rating scales.

Nevertheless, the choice of rating scale format should not be based on reliability considerations alone. Previous research has shown that scales with a larger number of response categories may be more susceptible to response styles such as extreme responding or acquiescence (Baumgartner & Steenkamp, 2001; Preston & Colman, 2000). In addition, increasing the number of response options may impose greater cognitive demands on respondents and increase respondent burden (Krosnick & Presser, 2010). These factors may introduce additional sources of systematic error that were not examined in the present study. Therefore, the practical implications of the present findings should be interpreted in light of the specific research context, measurement goals, and characteristics of the target population.

It should also be noted that the present study focuses specifically on the methodological consequences of discretizing continuous responses within traditional analytical frameworks. Other potential influences on reliability, such as response styles or scale design features, were not examined in this study and warrant further investigation.

Several limitations should be noted. First, the present study focused on reliability and did not examine other factors relevant to scale design, such as response styles or respondent burden. Second, the empirical findings were based on a single instrument administered to Taiwanese junior high school students, and the observed breakpoint pattern may therefore vary across constructs, populations, and assessment contexts. Third, although the breakpoint analysis provided a formal estimate of where marginal gains began to diminish, this estimate should not be assumed to be identical across all measurement settings. Future research should examine whether similar patterns emerge across other constructs, samples, and testing contexts.

In conclusion, this research provides new empirical evidence regarding how response granularity relates to reliability in psychological measurement. Across both simulation and empirical data, continuous response formats yielded the highest reliability, confirming their theoretical advantage in capturing subtle individual differences. However, the empirical breakpoint analysis indicated that the rate of improvement diminished substantially after approximately seven to eight response options within the discrete range examined here, even though highly fine-grained and continuous formats continued to yield the highest estimates.

The findings also advance a unified perspective on scale granularity, framing discrete and continuous scales as points along a single continuum rather than categorically distinct types. This view offers a potential explanation for some of the inconsistencies reported in previous studies. Methodologically, the study demonstrates the value of CoRSA as an IRT-based approach for directly estimating reliability from continuous data without discretization (Chou et al., 2025). Together, these insights provide a more well-defined theoretical and practical foundation for scale design, suggesting that moderate-to-fine discrete formats may serve as efficient practical options, whereas very fine-grained and continuous formats remain preferable when maximizing reliability is the primary goal.

## Data Availability

The datasets analyzed in the present study are available in the Open Science Framework repository at https://osf.io/vq6xg

## Code availability

The program code used in this research are available in the Open Science Framework repository at https://osf.io/vq6xg
